# Midodrine and Weekly Albumin Therapy in Patients With Cirrhosis and Diuretic Intractable or Recurrent Ascites: A Case-Control Study

**DOI:** 10.7759/cureus.76988

**Published:** 2025-01-06

**Authors:** Gourav J Borah, Gaurav Pande, Sayan Malakar, S Rakesh Kumar, Rajanikant R Yadav, Samir Mohindra

**Affiliations:** 1 Department of Gastroenterology, Sanjay Gandhi Postgraduate Institute of Medical Sciences, Lucknow, IND; 2 Department of Radiodiagnosis, Sanjay Gandhi Postgraduate Institute of Medical Sciences, Lucknow, IND

**Keywords:** albumin, diuretic intractable ascites, midodrine, mortality, recurrent ascites

## Abstract

Background

Overwhelming splanchnic and systemic vasodilatation and low mean arterial pressure (MAP) pose significant challenges in mobilizing ascites in patients with decompensated cirrhosis. We aimed to evaluate the efficacy and survival benefits of oral vasoconstrictor and weekly albumin therapy in patients with diuretic intractable and recurrent ascites in cirrhosis.

Materials and methods

A total of 113 cirrhotic patients with diuretic intractable (n=45) and recurrent ascites (n=68) with MAP ≤ 82 mmHg were included. Of the 113 patients, 85 patients received midodrine (12.5±2.5 mg thrice daily) and weekly albumin 20-40 g/day to achieve a serum albumin level of 4 g/dL with standard medical therapy (SMT), whereas 28 patients received SMT alone. On follow-up, at three months, we evaluated and compared the control of ascites, urinary sodium, systemic vascular resistance (SVR), and renal arterial resistive index (RARI) between arm 1 and arm 2.

Results

Among 113 patients, mean Child-Turcotte-Pugh (CTP) and Model for End-stage Liver Disease-sodium (MELD-Na) scores were comparable in both arms (CTP 9.52±1.16 and 9±1.18 and MELD 21.33±4.17 and 22.36±4.2 in arm 1 and arm 2, respectively). After three months of therapy in arm 1, a significant increase was seen in urinary sodium excretion (25.99±15.73 and 114.38±71.33 meq/24 hours), MAP (78.91±3.11 and 84.3±3.13 mmHg), SVR (1,059.4±23.09 and 1,178.3±12.39 dynes/s/cm⁵), and decrease in RARI (0.71±0.054 and 0.67±0.039) (p <0.05). The median overall survival and ascites control were better in arm 1 (p <0.001) at the end of follow-up (12 months).

Conclusion

Response-guided midodrine and weekly albumin therapy, along with SMT, have better overall survival and ascites control.

## Introduction

Ascites is the most common complication of portal hypertension in patients with cirrhosis. It is conventionally managed with diuretics and dietary sodium restriction of <2 g/day [1]. However, despite adhering to standard treatment, management of ascites in a large number of patients is difficult [2]. Diuretic-intractable ascites occurs in approximately 5% to 10% of all ascites cases [3]. Of these patients, 50% die within six months of development [4]. Splanchnic vasodilation, decreased effective blood volume, reduced renal blood flow, and renin-angiotensin-aldosterone system (RAAS) activation are critical factors in the pathogenesis of functional renal abnormalities and, thereby, ascites in patients with cirrhosis. Concomitantly, low mean arterial pressure (MAP) and high renal arterial resistive index (RARI) due to local factors (RAAS, sympathetic nervous system activation), leading to low glomerular filtration rate (GFR), make it more difficult to mobilize ascites [4,5]. Vasoconstrictor administration decreases systemic arteriolar vasodilation and improves MAP, thereby increasing renal blood flow [5,6].

Midodrine hydrochloride, an alpha-1 agonist that directly acts on peripheral alpha-receptors, is an oral vasoconstrictor [7]. Systemic and renal hemodynamics in cirrhotic individuals with hepatorenal syndrome have been demonstrated to significantly improve upon administration of midodrine [8]. However, clinical trials using midodrine in patients with liver cirrhosis-related ascites have yielded inconsistent results, and hemodynamic alterations have not been examined previously.

Administration of albumin with diuretics in patients with ascites is associated with improved survival; however, little is known about the long-term use of albumin, particularly when combined with midodrine. Along with its oncotic function, albumin may also have antioxidant effects, improvement of endothelial function, and immunomodulatory effects [9]. These properties may be helpful in preventing cirrhosis-related complications. Albumin is known to prevent post-paracentesis circulatory dysfunction (PPCD) following large-volume paracentesis (LVP); however, there is a dearth of research on the effects of albumin on systemic and renal hemodynamics [10].

Ascites becomes more refractory to treatment as MAP is reduced to <82 mmHg and RARI increases. Adding a vasoconstrictor early may help correct the above abnormality and improve GFR and RARI, thereby facilitating a better diuretic action [11]. Since furosemide is >95% bound to albumin, and, thus, apart from its oncotic effect, the diuretic binding effect of albumin may be augmented if serum albumin is high [12].

In this context, this study was conducted to evaluate the efficacy and survival benefit of response-guided therapy with midodrine combined with weekly albumin in diuretic intractable or recurrent ascites in patients with cirrhosis.

## Materials and methods

In this prospective, open-label, non-randomized, case-control study, a total of 113 patients of cirrhosis with recurrent or diuretic intractable ascites attending the outpatient department (OPD) of the Department of Gastroenterology, Sanjay Gandhi Postgraduate Institute of Medical Sciences, Lucknow, India, were enrolled. This study complied with the Declaration of Helsinki and was approved by the Institutional Ethics Committee (IEC 2023-113-DM-131).

Cirrhosis was diagnosed based on history, physical examination, and radiological investigations with or without liver biopsy [13]. Recurrent ascites was defined as grade 3 ascites that recurred on at least three occasions within 12 months despite standard treatment [14]. Diuretic intractable ascites was defined as ascites that could not be mobilized or the early recurrence of which could not be prevented because of the development of diuretic-induced complications that preclude the use of an effective diuretic dose [15]. Early ascites recurrence was defined as the re-appearance of grade 2 or moderate ascites with moderate symmetrical abdominal distention, or grade 3 with massive ascites with marked abdominal distention within four weeks of initial mobilization [16]. LVP with an infusion of albumin was performed in both arms for tense, symptomatic ascites despite maximal tolerable diuretic therapy or inability to use an effective dose of diuretics owing to diuretic-related side effects [14-17]. All the patients were instructed to come for paracentesis when they became symptomatic.

At enrollment, a baseline workup was performed, including body weight, blood pressure, heart rate, liver function tests, renal function tests, urinary sodium, electrocardiogram, systemic vascular resistance (SVR), and RARI. Patients were asked to measure their daily urine output at the time of enrollment and at the end of therapy. SVR was measured as (MAP − mean right atrial pressure [RAP]) × 80 / cardiac output using 2D echocardiography [18]. RARI was calculated by the Doppler ultrasound using the formula: RARI = (peak systolic velocity - end-diastolic velocity) / peak systolic velocity. Three values were calculated for both the right and left kidneys, and the mean value was used to minimize intra-observer errors.

**Figure 1:**
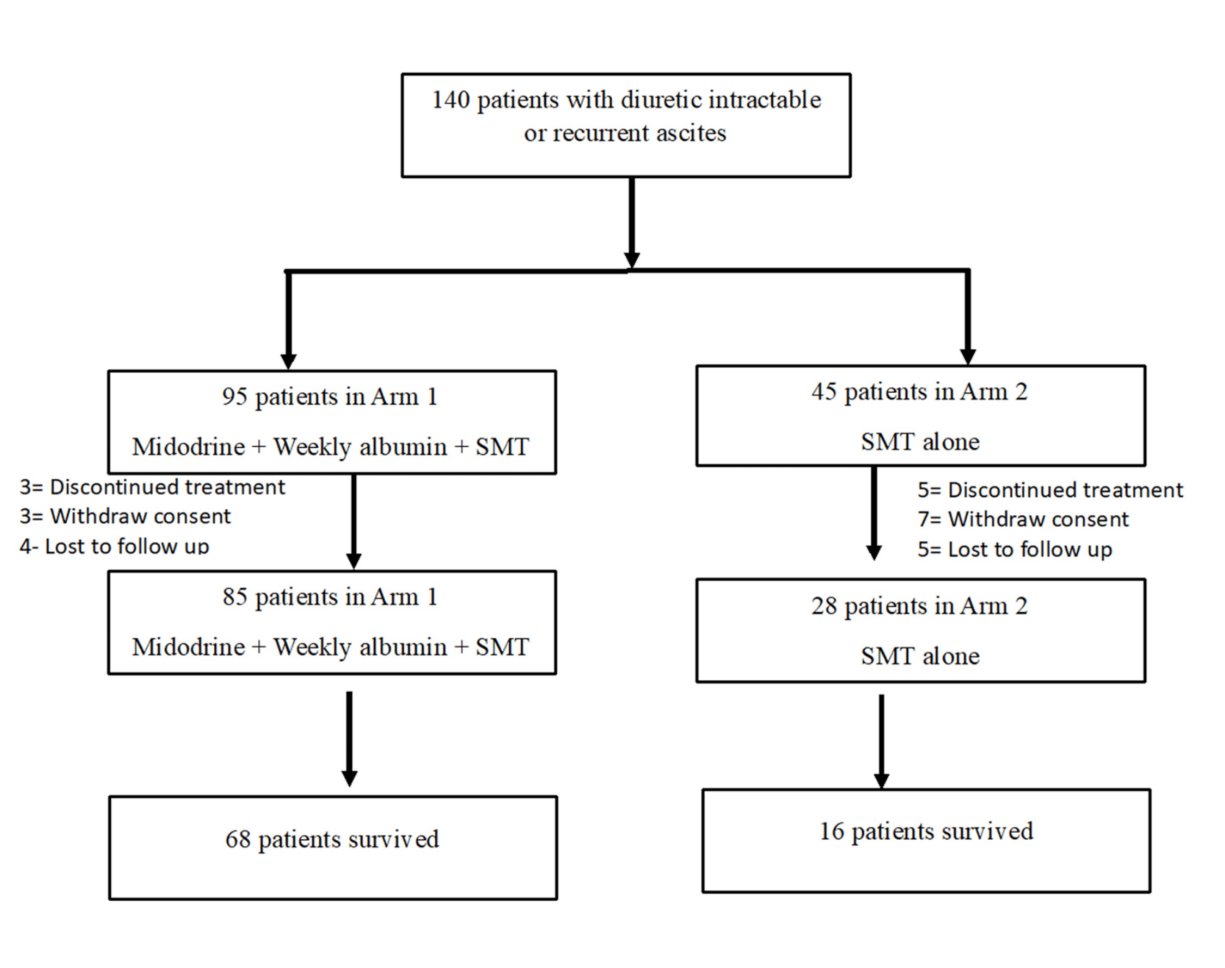
Consort diagram showing the number of subjects enrolled in the study. SMT, standard medical therapy

Midodrine is related to increased urine output and ascites mobilization in refractory ascites, as demonstrated in a previous study by Singh et al.; therefore, patients were included in arm 1 and arm 2 at a 2:1 ratio in this study [6]. A total of 140 patients were divided into two arms. Arm 1 included 95 patients receiving midodrine (12.5±2.5 mg thrice daily) and weekly albumin therapy (20-40 g/week to achieve serum albumin level of 4 g/dL) along with standard medical therapy (SMT). Midodrine was started at a 5-mg thrice-daily dose and titrated gradually. After achieving ascites mobilization, the dose was reduced to maintain a MAP of >82 mmHg. The MAP goal of 82 mmHg was kept, as prior studies have demonstrated that patients with ascites who attained MAP > 82 mmHg had improved survival rates [19]. Ten patients discontinued treatment in arm 1; therefore, a total of 85 patients were included for analysis. Arm 2, which consisted of 45 patients, received SMT only. SMT was defined as dietary salt restriction (<5 g/day), LVP, and/or diuretic use. LVP was performed along with intravenous albumin (8 g/L of ascites removed) using standard clinical methodology, wherever required. In this arm, 17 patients discontinued treatment or were lost to follow-up; hence, a total of 28 patients were considered for analysis (Figure 1). Kidney Disease Improving Global Outcomes (KDIGO) guideline was used to define acute kidney injury. The clinical and biochemical parameters were assessed in all patients at baseline and after three months. Patients were followed up in the OPD at two-week intervals until MAP was >82 mmHg and urinary sodium was >80 mmol/L and then at monthly intervals until death or termination of the study. For those who did not respond to the treatment, transjugular intrahepatic portosystemic shunt (TIPS) placement and repeated LVP were advised.

Inclusion and exclusion criteria

Adult patients (>18 years) with diuretic intractable or recurrent ascites and low MAP (<82 mmHg) were included in our study. Patients with gastrointestinal bleeding, infection within one month preceding or during the study, hepatic encephalopathy of grade 2 or higher, intrinsic renal disease, heart failure, coronary artery disease, hepatocellular carcinoma, and portal vein thrombosis were excluded. Patients who received midodrine or albumin four weeks prior to the study were also excluded. Those who did not give consent and were less than 18 years of age were excluded from this study.

Outcome

The primary outcome measure was ascites mobilization in both arms (grade 1 or no ascites on ultrasonography). Secondary outcome measures were survival in both arms and adverse events.

Endpoint

The endpoint of the study was death or the completion of 12 months of follow-up.

Statistical analysis

Mean ± standard deviation (SD) was used for continuous variables with normal distribution, while frequency/percentage was used for categorical data. The independent samples t-test or Mann-Whitney U-test was used for comparison between means/medians of the two arms, while the chi-squared test was used to compare proportions. A paired t-test was used to compare the means between pre- and post-treatment observations. The Kaplan-Meier method with the log-rank test was used to compare the survival distributions between the two arms. SPSS Version 26 (IBM Corp., Armonk, NY, USA) and MedCalc software were used for the data analysis. The results were reported as mean values ± SD. The level of significance was established at 0.05.

## Results

Among the 113 patients enrolled in this study, the mean age was 47.19±10.12 years (range: 39-55 years), with predominantly male patients (78.8%). The mean ages of patients in arm 1 and arm 2 were 46.6±10.37 and 49±9.23 years, respectively (p = 0.393). The baseline clinical, demographic, and biochemical characteristics of the patients were comparable between the two arms (Table 1). Alcohol was the most common etiology of cirrhosis in both groups (38.9%). This was followed by hepatitis C, hepatitis B, and autoimmune hepatitis, which accounted for 9.7%, 8.8%, and 5.3% of cases, respectively (Table 2). Mean Child-Turcotte-Pugh (CTP) and Model for End-stage Liver Disease (MELD) scores were 9.52±1.16, 21.33±4.17 and 9±1.18, 22.36±4.2 in arm 1 and arm 2, respectively (p = >0.05), which were comparable. In both treatment arms, no significant difference in weight was noted at baseline. However, the change in body weight was significant in the midodrine and weekly albumin arm at the end of three months (p = <0.003).

**Table 1:** Distributions of the demographic and clinical variables between two arms. CTP, Child-Turcotte-Pugh; MELD, Model for End-stage Liver Disease; UO, urine output; UNa, urinary sodium; MAP, mean arterial pressure; S., serum; INR, international normalized ratio; T. bilirubin, total bilirubin Data are presented as mean±SD

Variables	Total (n=113), mean±SD	Arm 1 (n=85), mean±SD	Arm 2 (n=28), mean±SD	p-Value	T-value
Age (years)	47.19±10.12	46.6±10.37	49±9.23	0.393	-0.336
CTP	9.39±1.54	9.52±1.16	9±1.18	0.164	1.444
MELD	21.58±4.19	21.33±4.17	22.36±4.2	0.37	-0.526
S. creatinine (mg/dL)	1.5±1.06	1.59±1.17	1.22±0.5	0.458	0.753
S. Na (mmol/L)	130.18±7.25	130.12±7.62	130.36±6.11	0.458	-0.753
Weight loss (kg)	5.2±8.2	11.11±5.39	-.571±3.3	<0.003	3.760
UO at baseline (mL)	607.96±252.63	587.06±273.89	671.43±160.69	0.084	-1.368
UNa (mmol/L) at baseline	23.55±14.35	25.99±15.73	16.14±2.82	0.001	0.993
UNa (mmol/L) at 3 months	97.58±68.66	114.38±71.33	46.5±11.8	0.001	4.410
MAP (mmHg) at baseline	78.6±3.04	78.91±3.11	77.79±2.68	<0.001	2.011
MAP (mmHg) at 3 months	82.5±4.33	84.3±3.13	76.93±2.01	<0.001	7.729
S. albumin (g/dL)	2.42±0.42	2.38±0.45	2.54±0.3	0.051	-1.415
INR	2.2±0.53	2.21±0.58	2.19±0.37	0.842	-0.183
T. bilirubin (mg/dL)	2.76±0.81	2.8±0.83	2.65±0.79	0.415	1.644
Follow-up (days)	289.88±119.79	338.05±83.72	143.64±90.74	<0.001	4.434

**Table 2:** Aetiology of cirrhosis AIH, autoimmune hepatitis; HBV, hepatitis B virus; HCV, hepatitis C virus

Etiology	Arm 1 (n=85), numbers (percentage)	Arm 2 (n=28), numbers (percentage)	Total (n=100), numbers (percentage)
AIH	4 (4.7)	2 (7.1)	6 (5.3)
Alcohol	33 (38.9)	11 (39.3)	44 (38.9)
Cryptogenic	32 (37.6)	10 (35.7)	42 (37.2)
HBV	7 (8.2)	3 (10.8)	10 (8.8)
HCV	9 (10.6)	2 (7.1)	11 (9.7)
Total	85 (100)	28 (100)	113 (100)

Baseline serum creatinine was 1.59±1.17 mg/dL in arm 1 compared to 1.22±0.5 mg/dL in arm 2 (p = 0.458). Baseline MAP did not differ between the SMT and midodrine + albumin group (p = 0.704). A significant increase in MAP was noted in the midodrine group (78.91±3.11 and 84.3±3.13 mmHg at baseline and at the end of three months, respectively) (p = 0.001). However, it did not change significantly in the SMT group (77.79±2.68 vs 76.93±2.01). At the end of three months, urine output was significantly higher in arm 1 after treatment (p = 0.001) but not in arm 2. A significant increase in urinary sodium excretion was noted (114.38±71.33 vs 46.5±11.8 meq/24 hours in arm 1 and arm 2, respectively; p = 0.001) at the end of three months in the midodrine and weekly albumin group compared to the SMT alone group. There was a significant increase in SVR (1059.4±23.09 to 1178.3±12.39 dynes/s/cm⁵; p = 0.00) and a decrease in RARI (0.71±0.054 and 0.67±0.039; p = 0.0002) at the end of three months in arm 1 as compared to arm 2 (SVR 1,083.5±29.17 to 1,077.3±38.59, p = 0.72 and RARI 0.70±0.03 to 0.71±0.03, p = 0.09) (Table 3).

**Table 3:** SVR and RARI in both arms SVR, systemic vascular resistance; RARI, renal arterial resistive index Data are presented as mean ± SD

	Arm 1 at baseline	Arms 1 at 3 months	p-Value	T-value	Arm 2 at baseline	Arms 2 at 3 months	p-value	T-value
SVR (dynes/s/cm⁵)	1,059.4±23.09	1,178.3±12.39	0.00	-18.05	1,083.5±29.17	1,077.3±38.59	0.72	1.87
RARI	0.71±0.054	0.67±0.039	0.0002	11.5	0.70±0.03	0.71±0.03	0.09	-1.72

Ascites mobilization

Midodrine therapy was superior to SMT alone in controlling ascites, with a complete response in 78.8% of patients (control of ascites in arm 2: 32.14%; p = 0.006). More LVP was required in arm 2 than in arm 1 (10.6% in arm 1 vs 82.1% in arm 2; p ≤ 0.002). The required diuretics dosage was also lower in arm 1 (furosemide 40±20 mg and spironolactone 50±25 mg in arm 1 vs furosemide 80±20 mg and spironolactone 100±25 mg in arm 2) (Table 4).

**Table 4:** Outcome in both arms LVP, large-volume paracentesis

Outcome	Arm 1 (n=85)	Arm 2 (n=28)	pValue	Chi-square value	T-value
Survival at 12 months (%)	80	57	0.001	13.6	-
Median survival (months)	12.81	8.09	0.000	-	7.7
Number of LVP	0.58±0.31	5.29±2.9	0.0001	-	-8.81
Complete ascites mobilization (%)	78.8	32.14	0.006	12.9	-

Survival

**Figure 2:**
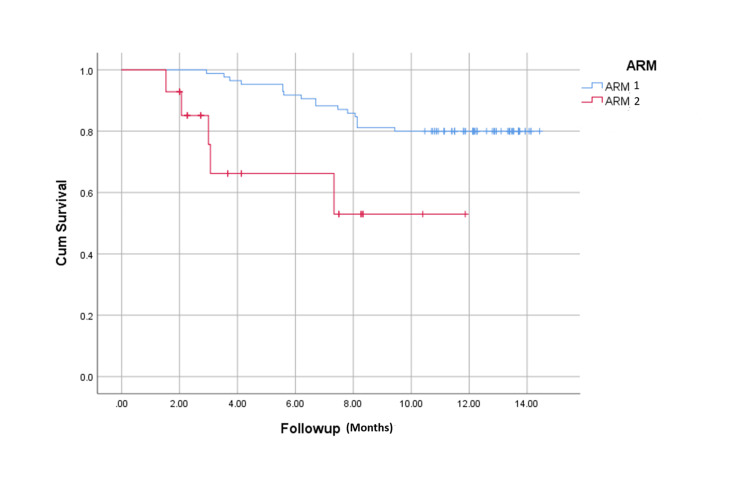
Kaplan-Meier analysis showing overall survival probability of patients in response-guided midodrine and weekly albumin arm (arm 1) versus standard medical therapy arm (arm 2).

**Figure 3:**
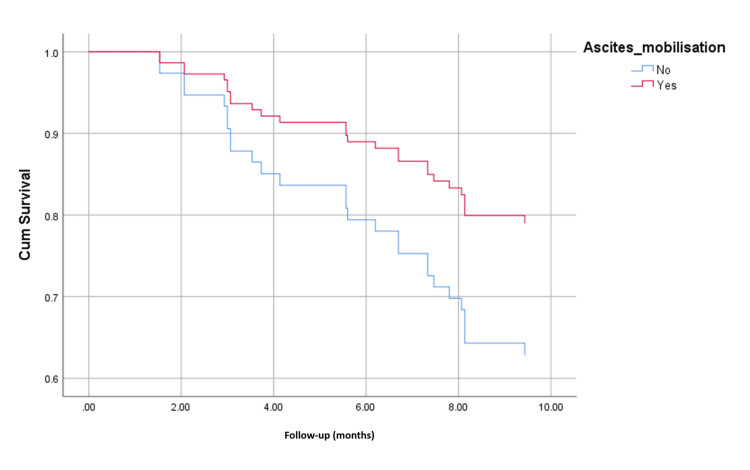
Kaplan-Meier analysis showing survival probability of patients in ascites mobilized patients versus those who did not.

**Figure 4:**
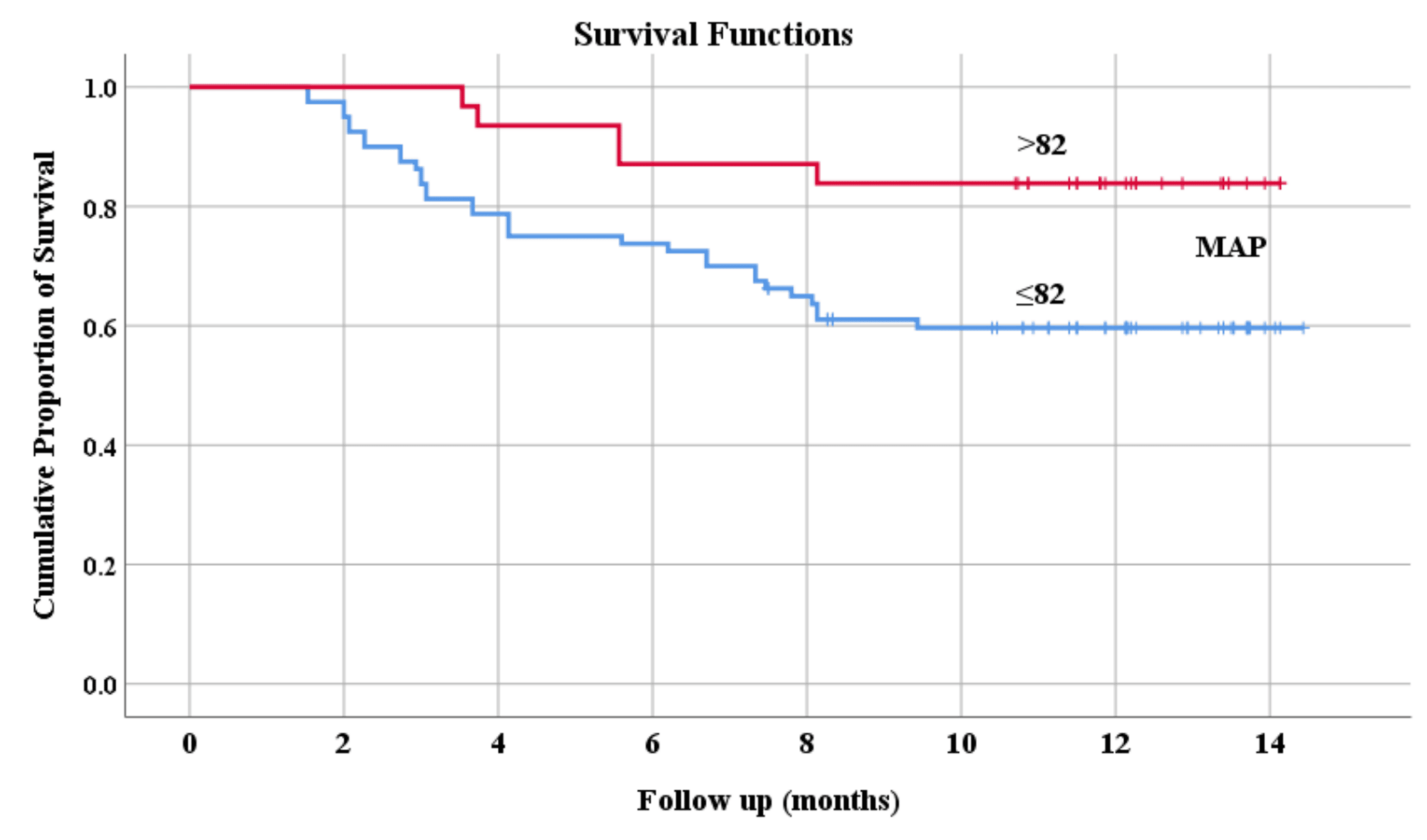
Kaplan-Meier analysis showing survival probability of patients with MAP > 82 mmHg versus ≤ 82 mmHg. MAP, mean arterial pressure

The overall survival rates were 80% (68 of 85 patients) in arm 1 and 57% (16 of 28 patients) in arm 2 during the follow-up period of 289.88±119.79 days (Figure 2). Mean survival in arm 1 and arm 2 were 12.81 and 8.09 months (p = 0.000), respectively. Overall mortality was lower in patients whose ascites were mobilized (32.5% vs 67.5%; p = 0.004) (Figure 3). Those who maintained MAP > 82 mmHg had a higher survival probability (Figure 4).

Follow-up

Overall, 20 patients in arm 1 required hospital admissions compared to 24 patients in arm 2. Five and three non-responders underwent TIPS in arm 1 and arm 2, respectively. The other non-responders refused to undergo TIPS.

Adverse events

Four patients in arm 2 experienced muscle cramps, while 23 patients in arm 1 complained of urgency or urinary retention. Nine patients in arm 1 and 10 in arm 2 had hyponatremia (serum sodium level < 130 mmol/L). Among patients in arm 2, local site bleeding, ecchymosis, acute kidney injury, and spontaneous bacterial peritonitis (SBP) were significantly higher (Table 5).

**Table 5:** Adverse events in both arms Hyponatremia is taken as serum sodium < 130 meq/L.

Adverse events	Arm 1 (n=85)	Arm 2 (n=28)	p-Value	Chi-squared value
Hyponatremia	9	10	<0.001	52.8
Hypokalemia	10	3	<0.001	43.18
Urinary retention or urgency	23	0	<0.001	-
Hepatic encephalopathy	5	8	0.64	0.219
Muscle cramps	7	4	0.001	14.00
Local site bleeding	4	7	<0.01	6.22
Hematoma/ecchymosis	0	4	<0.01	-
Spontaneous bacterial peritonitis	5	6	0.001	21.66
Acute kidney injury	2	5	<0.01	9.9
Gastrointestinal bleed	14	9	0.59	3.57

## Discussion

In this case-control study, we examined the impact of hemodynamic correction in patients with diuretic intractable or recurrent ascites. Compared to the standard treatment arm (arm 2), arm 1 showed improved MAP, higher SVR, and lower RARI. Elevated urinary sodium excretion and daily urine production were associated with lower requirement for LVP and better ascites control in these patients. A total of 78.8% of patients in arm 1 had complete ascites mobilization, and these patients had improved overall survival rates. Less acute kidney injury and hyponatremia were observed in arm 1 compared to arm 2, although there were higher reports of muscle cramps and urinary urgency in arm 1. Arm 2 had a higher incidence of SBP.

The therapeutic options available for patients not responding to diuretics are serial therapeutic LVP, TIPS, and liver transplantation [1,4]. However, repeated paracentesis itself can cause many complications, including PPCD, SBP, and hematoma. [15]. Thus, another therapeutic option - the role of vasoconstrictors in this group of patients with diuretic-intractable or recurrent ascites - is increasingly studied [6,8]. In patients with large ascites with hepato-renal syndrome-acute kidney injury (HRS-AKI), the addition of vasoconstrictors such as terlipressin or noradrenaline with albumin is the standard of care [20,21]. A previous study from our center showed that response-guided treatment with vasoconstrictors, albumin, and furosemide to maintain MAP >82 mmHg had better ascites control and improvement in AKI in acute on chronic liver failure patients [11]. However, many find injectable vasoconstrictors in ambulatory settings onerous. As a result, oral vasoconstrictors such as midodrine may play a significant role in difficult-to-treat ascites. The rationale for treating patients with midodrine and weekly albumin therapy was that both medications improved systemic and renal hemodynamics. Midodrine increases effective arterial blood volume by causing splanchnic vasoconstriction and improving renal perfusion and glomerular filtration [22]. Our earlier study indicated that individuals with large ascites had a higher RARI, which is comparable to the findings of this study. Response-guided hemodynamic improvement was observed with a reduction in RARI and an increase in SVR culminating in increased urinary salt excretion, urine output, weight loss, and better ascites mobilization in this study [11]. Midodrine, along with octreotide and albumin, has been shown to improve ascites control in a short-term pilot study including patients with refractory ascites [23]. In a similar prospective study of 40 cirrhotic patients with refractory or recurrent ascites, long-term administration of midodrine and SMT was associated with significantly decreased cardiac index and heart rate and increased MAP, SVR, and GFR [6]. Kalambokis et al. in their study on the effects of a seven-day treatment with midodrine in patients with cirrhosis reported that it improved systemic hemodynamics and increased urinary sodium excretion. These were associated with suppression of RAAS, which further suggested improvements in effective arterial blood volume and thereby improvements in urinary sodium excretion [24]. In line with our results, Rai et al. reported a significant increase in urine volume and urinary sodium in the midodrine group at one and three months [25]. Another randomized controlled trial (RCT) showed a substantial reduction in body weight and abdominal girth after two weeks of midodrine therapy [26]. At three months of therapy, the midodrine group had a significantly higher incidence of response to treatment in the form of complete ascites mobilization (78.1%). Singh et al. showed that 94% of patients in the midodrine arm and 50% of patients in the control arm achieved complete or partial control of ascites at three months, which is consistent with our findings [6]. This study found that decreased complication rates were associated with lower dosages of diuretics required for ascites control, implying a possible role of response-guided oral vasoconstrictors in these patients.

Our findings demonstrated that response-guided treatment with midodrine and weekly albumin enhanced overall survivability. The benefits of mortality in arm 1 may probably be related to improvements in MAP and SVR, and a reduction in RARI. In the ANSWER trial, survival benefits were observed with albumin, but other RCTs did not show any benefits of albumin administration [27-29]. In the MACHT trial including 196 consecutive patients with cirrhosis and ascites awaiting liver transplantation, no significant differences in the developing complications (p = 0.402) or one-year mortality (p = 0.527) were observed during follow-up [28]. This may be due to advanced liver disease in the treatment arm, a shorter duration of treatment (median 80 days), and a lack of response-guided therapy, as shown in our study. Our data revealed a positive correlation between ascites mobilization, high MAP, and survival. This could again be connected to improved hemodynamics in the treatment arm (arm 1) following the intervention. However, more robust randomized controlled studies are required to confirm these results.

Our study's strength is that it is the only study to evaluate renal and systemic hemodynamics as well as survival in patients with diuretic intractable or recurring ascites. Extended patient follow-up was the second strength of the present study.

The shortcomings of our study include its open-label, observational, non-randomized, single-center design. The smaller number of patients in the standard treatment group rendered these results less robust. Since the serum aldosterone level and plasma renin activity were not performed at our institution, these parameters were not measured in this study.

## Conclusions

The findings of this study imply that response-guided midodrine and weekly albumin therapy, when combined with SMT, improve patients' overall survival, ascites control, and fewer complications in cirrhosis patients. MAP values greater than 82 mmHg and ascites mobilization following therapy were associated with improved survival. To validate these results, larger RCTs are needed in the future.
